# Racket Sports-Related Injuries in Youth Athletes: A Narrative Review

**DOI:** 10.3390/ijerph23010135

**Published:** 2026-01-22

**Authors:** Mahesh Shrestha, Asra Usmani, Serena Karlov, Ann Harris, Dilip R. Patel

**Affiliations:** 1 Department of Pediatric and Adolescent Medicine, Western Michigan University Homer Stryker M.D. School of Medicine, 1000 Oakland Drive, Kalamazoo, MI 49008, USA; asra.usmani@wmed.edu (A.U.); dilip.patel@wmed.edu (D.R.P.); 2 Western Michigan University Homer Stryker M.D. School of Medicine, 300 Portage Street, Kalamazoo, MI 49008, USA; serena.karlov@wmed.edu; 3 Department of Medical Library, Western Michigan University Homer Stryker M.D. School of Medicine, 300 Portage Street, Kalamazoo, MI 49008, USA; ann.harris@wmed.edu

**Keywords:** racket sports, pediatrics, adolescents, injuries, sport injuries, overuse injuries

## Abstract

Objectives: The popularity of racket sports has been increasing globally in recent years, with tennis remaining the most played sport worldwide and pickleball gaining popularity in the United States. While many studies have addressed injuries associated with racket sports in adult athletes, minimal data are available focusing on young athletes in pediatric and adolescent populations. This study aimed to review the various injuries associated with racket sports in pediatric and adolescent populations. Methods: Using the PubMed database, we searched for studies completed in the last 10 years that addressed injuries due to racket sports in age groups up to 18 years old, as well as adult studies that included pediatric and adolescent populations. Results: In total, 60 studies met our inclusion criteria, of which 18 were descriptive studies. The most common injuries reported in the studies were lower extremity injuries, specifically knee and ankle injury. Upper extremity injuries were the next most common, specifically shoulder injuries. Most of the studies reported multiple musculoskeletal injuries as opposed to just one. Tennis was found to be the racket sport that caused the highest number of injuries, as well as the most severe injuries. Conclusions: There are few studies on musculoskeletal injuries from racket sports in pediatric and adolescent populations. This review found that tennis had the highest number of studies, and different types and severities of injuries were well-described. Lower extremity overuse injuries were the most common, followed by upper extremity injuries. Eye injuries were less common but serious. Injuries cause pain, time loss in school, and increased healthcare burden, so there is public health relevance to conducting more racket sport studies. There is a significant amount of physical and mental growth occurring during childhood and adolescence; therefore, more kinematic studies and systematic reviews should be conducted pertaining to racket sports, which will hopefully help with injury prevention in these age groups.

## 1. Introduction

Racket sports involve the use of a racket and ball. A racket can be a hollow bat interwoven with strings, as in badminton and tennis, or it can be a solid bat, as in pickleball and table tennis. While tennis, badminton, squash, and table tennis are popular racket sports, there are more than 30 different racket sports which could be grouped as (a) tennis and variants—tennis, real tennis, soft tennis, platform tennis, paddle tennis, padel, squash tennis, tennis polo, and sticke tennis; (b) badminton and variants—badminton, table tennis, ball badminton, and speedminton/crossminton; (c) squash and variants—squash, hardball squash, racquetball, racketball, and racquets; (d) pickleball and variants—pickleball, pitton, paleta fronton, matkot/frescobol, pelota mixteca, beach tennis, speedball, qianball, racketlon, and tamburello; and (e) pelota and variants—Basque pelota, fives, frontenis, and Jai alai [1].

Racket sports, including tennis, badminton, squash, pickleball, and padel, have witnessed a significant surge in global popularity in recent years. Tennis continues to be a widely played sport worldwide with substantial participation across various age groups. Badminton and squash also have strong global followings, with millions of people engaging in these sports recreationally and competitively. Padel, a hybrid of tennis and squash, has emerged as one of the fastest-growing sports globally, with over 25 million players in 90 countries [2]. In the United States, where tennis and squash have always been common racket sports for many years, pickleball, which uses a small bat with a plastic ball with holes, has experienced an unprecedented rise in popularity. According to the Sports & Fitness Industry Association’s 2025 Topline Participation Report, pickleball participation reached 19.8 million in 2024, marking a 45.8% increase from that in a year. This rapid growth has been particularly notable among older adults, with most players being over the age of 50, though it also acknowledges that the sport is popular across all ages, including children and adolescents [3,4].

Traditional racket sports, such as tennis, badminton, and squash, have always been popular and offer various health benefits. However, due to their fast action, they could cause various injuries, such as sprains and fractures, including the commonly known ‘tennis elbow’. Lambert et al. [5] performed a cross-sectional study focused on common racket sport injuries involving tennis, badminton, and table tennis within one Olympic cycle and found that 55% out of 390 athletes suffered a serious injury, and 78% of the most common injuries involved the lower extremities. The surge in the popularity of pickleball has been accompanied by a corresponding increase in musculoskeletal injuries. A study analyzing data from the U.S. The Consumer Product Safety Commission’s National Electronic Injury Surveillance System (NEISS) between 2013 and 2022 found a significant rise in pickleball-related injuries, particularly among individuals aged ≥60 years. These injuries predominantly involve the upper and lower extremities, with common diagnoses including sprains, strains, and fractures [6,7].

While injury patterns in adult racket sport athletes have garnered attention, there remains a notable scarcity of research focusing on young athletes in pediatric and adolescent populations. We started with a search of studies involving children and adolescents with pickleball injuries, and when we could only find a single study on pickleball, we decided to conduct a review study of all racket sport injuries, as we found a lack of review studies in this age group. A study of injuries in youth athletes, including children and adolescents, due to racket sports is important because they are susceptible to unique injuries because of growth in the musculoskeletal system, including injuries to the growth plate, apophysis, and joint surfaces [8,9,10]. There is also a limitation of studies involving injury surveillance, which could help quantify injury rates and evaluate prevention initiatives [11]. Understanding the causation of injuries and their prevention could help in forming public health guidelines for injury prevention and surveillance. Another benefit is the reduction in injury time, school absence, and hospitalization. This study aimed to describe all injuries related to racket sports published in adolescents in the last 10 years and classify these injuries based on location and type of racket sport.

## 2. Materials and Methods

We conducted a literature search in PubMed for articles related to racket sport injuries in pediatric and adolescent populations published between 2015 and 2025. Searching PubMed using the terms (“Racquet Sports”[Mesh] OR “Racquet Sports/injuries”[Mesh] OR “racquet sport*”[tiab] OR “racket sport*”[tiab] OR badminton[tiab] OR “ball badminton”[tiab] OR “battledore and shuttlecock”[tiab] OR crossminton[tiab] OR speedminton[tiab] OR frontenis[tiab] OR “lawn tennis”[tiab] OR qianball[tiab] OR rackets[tiab] OR racketball[tiab] OR racquetball[tiab] OR “real tennis”[tiab] OR “soft tennis”[tiab] OR speed-ball[tiab] OR squash[tiab] OR tennis[tiab] OR touchtennis[tiab] OR “table tennis”[tiab] OR “paddle tennis”[tiab] OR padel[tiab] OR pickleball[tiab] OR “platform tennis”[tiab] OR racketlon[tiab] OR sticke[tiab] OR “tennis polo”[tiab] OR “basque pelota”[tiab] OR “beach tennis”[tiab] OR “downside ball game”[tiab] OR “four wall paddleball”[tiab] OR frescotennis[tiab] OR jokari[tiab] OR matkot[tiab] OR kadima[tiab] OR frescobol[tiab] OR miniten[tiab] OR “one wall paddleball”[tiab] OR paddleball[tiab] OR “paddle ball”[tiab] OR “paleta fronton”[tiab] OR “pan pong”[tiab] OR “pelota mixteca”[tiab] OR pitton[tiab] OR “road tennis”[tiab] OR “spec tennis”[tiab] OR sphairee[tiab] OR stoolball[tiab] OR “table squash”[tiab] OR tamburello[tiab] OR “totem tennis”[tiab]) AND (“Athletic Injuries”[Mesh] OR “athletic injur*”[tiab] OR “sports injur*”[tiab] OR injur*[tiab]) AND (“Pediatrics”[Mesh] OR “Adolescent”[Mesh] OR “Child”[Mesh] OR child*[tiab] OR pediatric*[tiab] OR adolescent*[tiab] OR teen*[tiab] OR youth*[tiab]) returned 211 results in the last 10 years. Our inclusion criteria were (1) age group up to and including 18 years of age, but we also included adult studies that included pediatric and adolescents with separate findings for children; (2) studies that mentioned different injuries due to one or more racket sports in the last 10 years. Of the 211 studies identified in the PubMed search, we first examined their abstracts and then read the full texts to determine whether they met our criteria. Sixty studies met our inclusion criteria (Table S1). Common reasons for rejection were (a) search engine results that included studies on lacrosse as a racket sport, (b) adult studies, or (c) studies that only discussed the physiology of injury instead of the type of injuries. We divided the selected articles based on the type of study (case–control, systematic review, etc.), year of publication, location of injuries, and racket sport-specific injuries.

## 3. Results

**Figure 1:**
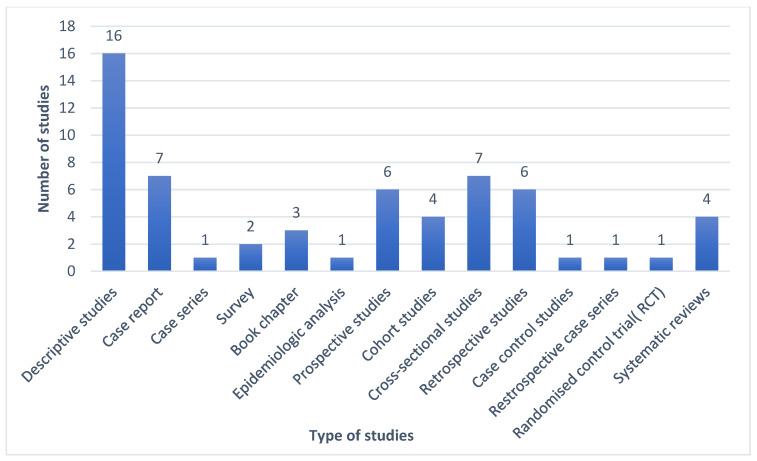
Distribution of type of studies by frequency.

Figure 1 shows the distribution of the study types by frequency. Of the 60 studies selected based on the criteria, most were descriptive studies, followed by cross-sectional studies and case reports.

**Figure 2:**
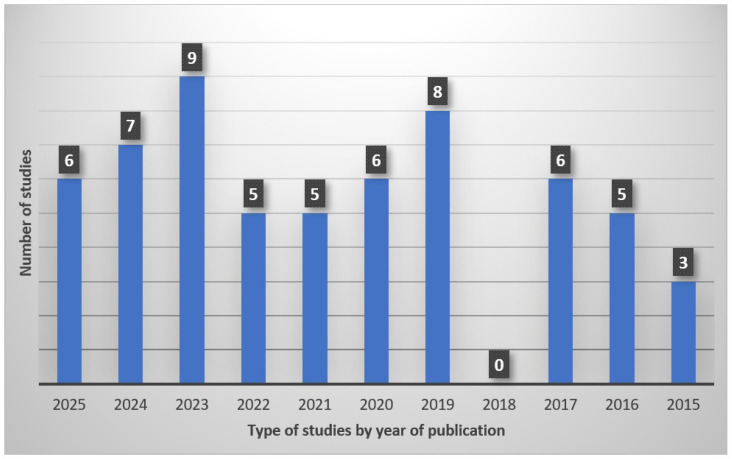
Distribution of studies by year of publication.

Figure 2 shows the distribution of studies by publication year. The highest number of studies published that met our criteria was in 2023, with nine publications that year. No studies that met our criteria were published in 2018, which could be due to publication patterns that year.

Table 1 provides an overview of the locations of injuries in the selected articles. Most injuries occurred in the lower extremities, specifically the knee and ankle. Shoulder injuries were the most common upper extremity injuries. There were a small number of traumatic eye injuries, but they were concerning in nature like traumatic hyphema, macular hole, etc. While some articles only described one injury, many articles described many different injuries; therefore, there is an overlap of injuries within this table. This table also highlights the different possible musculoskeletal injuries in racket sports. Since it does not differentiate which, specific injuries are more common with certain racket sports, we will take each racket sport and describe the injuries it causes.

We will now describe specific injuries related to certain common racket sports.

1. Tennis:
  Tennis is considered one of the most popular racket sports worldwide. Although tennis can have many health benefits in terms of good aerobic exercise and fitness, the dynamics involved in landing a powerful overhead serve with force generation from the hips, knees, and pelvis can lead to various types of injuries [12]. Thirty of the selected articles described some form of injury in tennis.
  1.1 Head and Eye injury: Patel et al. [13] studied tennis-related ocular injuries in the US from 2000 to 2019 with emergency department visits and found 16,000 tennis-related eye injuries, with males affected twice as much as females and the young age group (0–20 years) having the highest number of injuries, with most injuries in 11–15-year-old boys. One-third of hospitalized patients had open globe injuries. O’Connor et al. [14] studied the sport-related concussion (SRC) rate in high schoolers and found that the overall SRC rates per 10,000 exposures for boys and girls in tennis were 0.74 and 1.59, respectively. Girls collectively had a higher overall SRC rate than boys across all sports of 2.64 vs. 1.69, respectively.
  1.2 Upper extremity injury: There are many overuse injuries to the shoulder and elbow while playing tennis. Wang et al. [15] describe a case report of a youth athlete 14-year female Chinese semi-professional player with acute anterior rotator cuff strain. Caine et al. [16] performed a systematic review in young athletes and found that high-impact sports like tennis, badminton, etc., could cause periphyseal stress injuries in the shoulder, elbow, hand, wrist, foot and knee, and ankle and foot. Pasulka et al. [17] performed a case–control study in 1190 athletes and found that tennis, at 46.7%, was the sport with the highest proportion of single-sport-specialized athletes. Single-sport athletes in individual sports are specializing at a younger age (11.2 ± 2.4 vs. 12 ± 2.7, *p* = 0.05), and the authors reported higher training volumes than for team sports. Single-sport-specialized athletes in individual sports accounted for a higher proportion of overuse injuries (44.3% vs. 32.2%, *p* = 0.037) and serious overuse injuries (28.8% vs. 13.8%, *p* = 0.011) but a lower proportion of acute injuries (28.8% vs. 13.8%, *p* = 0.001) compared to single-sport athletes involved in team sports. Koyama et al. [18] reported a case report of stress fracture of scaphoid in an elite junior 18-year-old Japanese tennis player and concluded that it was due to repeatedly practicing an attacking backhand high volley, which involved too much dorsal flexion of the wrist. Young et al. [19] performed a cross-sectional study of 125 professional female athletes and reported a high level of infraspinatus atrophy in their dominant shoulders (age range of players: 17–36 years).
  1.3 Lower extremity: Holst-Christensen et al. [20] studied the mechanism of injury and return to sport rates following anterior cruciate ligament injuries in tennis among 231 patients and found that lunging, running forward to the net, and movements related to smashing were the most frequent activities leading to injury. Casadei and Kiel [21] reported that Little League shoulder, also called proximal humeral epiphysiolysis, which is common in baseball players and throwing athletes, is also observed in tennis players and competitive gymnasts. The typical age of presentation is between 11 and 16 years, with the mechanism of injury being the physis remaining open before the closure of the growth plate. Bittner and Hartstein [22] published a case report of fifth metatarsal avulsion fracture in a 17-year-old male tennis player. Brant et al. [23] conducted a descriptive epidemiology study from 2005 to 2016 using high school reporting information online (HSRIO) data and found that lower extremity sport injuries were higher in girls in tennis compared to boys, with rate ratios (RRs) of more than two to one. Girls had a higher proportion of severe lower extremity sport injuries needing imaging for all sports except volleyball. Ramponi and Baker [24] reported calcaneal apophysitis or Sever’s disease as the primary cause of heel pain in pediatric patients between 8 and 15 years from high-impact sports like tennis.
  1.4 Trunk and back injury: Gescheit et al. [25] performed a prospective cohort multiyear injury incidence study in elite junior Australian tennis players aged 13–18 years and found that the lumbar spine was the most commonly and severely injured area in both sexes, followed by shoulder and knee injuries. Whale et al.’s [26] data case report showed a 16-year-old Asian male who was an elite tennis player with one month history of left shoulder pain later found to have first rib stress fracture.
2. Badminton:
  In badminton, a softer racket than the one use in tennis is used, and a shuttlecock made of feathers rather than a harder ball like in tennis is used. Therefore, although badminton players exert a whip-like faster motion using their hand, causing faster head velocity, the use of a softer shuttlecock usually means less severe injuries [12]. Zhou et al. [27] studied epidemiological characteristics in 7–22-year-old badminton players from 2018 to 2023 and found that among 711 players, 60.3% suffered at least one badminton-related injury. The most common site was the knee (male 18.8%; female 18.6%), followed by the ankle and lower back. Saragaglia et al. [28] performed a retrospective epidemiological study of 135 patients between 10 and 66 years of age with acute badminton injuries and found that out of 146 total injuries, 88.3% were in the lower limbs, followed by 11% in the upper limbs and 0.7% in the head. In total, 61% were sprains, 22% were tendinous–muscular lesions, 9% were fractures, 2% were meniscal injuries and contusions, and there was one wound (0.7%). Zhou et al. [29] studied 366 badminton players aged 7–12 years via questionnaire and found that ankle pain was the most common, followed by knee, plantar, shoulder, and lower back. Lau and Mukherjee [30] studied shoulder and elbow injuries in 532 overhead youth athletes aged 12–18 years and other sports along with badminton. The prevalence of shoulder overuse injuries was 31%, and that of elbow overuse injuries was 9.2%. Being older (15–18 years), training >11 h per week, and having >8 years of experience increased the odds of injuries. Shaari et al. [31] performed a systematic review regarding metacarpal stress fracture in athletes (mean age 17 years) and found that badminton and tennis were the most common sports where players suffered metacarpal stress fracture and presented with pain in the dorsal hand with activity and recovered with return to play in 9 weeks following non-operative management. Jao et al. [32] and Yu et al. [33] described 12 (age range 16–77 years) and 85 patients (aged 15–65 years), respectively, with ocular injuries due to badminton. Among the 85 patients, 60 were hit by shuttlecocks and 25 were hit by their partners’ rackets. Eighty injuries were non-penetrating and five were penetrating. There were 58 cases of hyphema, 36 of secondary glaucoma, 23 of lens subluxation, and 2 of retinal detachment. There was long-term glaucoma-related morbidity in the 12-case series. Both studies recommend eye protection to reduce morbidity.
3. Pickleball:
  Pickleball is an increasingly popular sport worldwide, especially in the USA. Players use wooden rackets and hollow balls with holes, and a net, like in tennis, is used but the court is smaller. Only 1 out of 60 selected articles, the study by Boroumand et al. [34], described pickleball injuries, but it was a comprehensive overview of all upper and lower extremity injuries due to pickleball that were presented to emergency departments in the USA from 2003 to 2022 using the National Electronic Injury and Surveillance System (NEISS) database. There were 33 pediatric cases aged 12–15 years among the 749 patients (286 adults and 430 geriatric patients). Pediatric patients had more upper extremity injuries than adults (60.2% vs. 40.6%, *p* < 0.001 in children). Pediatric patients had a lower frequency of lower extremity injuries than adults (36.2% vs. 59.4%, *p* = 0.22). Pediatric patients also had a higher frequency of contusions than adults (18.2% vs. 5.9%, *p* = 0.020). Schools were the most common location for pediatric athletes (63.6%), followed by homes. Among pediatric injuries, 63.6% of injuries were in the upper extremity, and shoulder and finger injuries (each 18.2%) were the most common upper extremity locations, followed by the hand and wrist (9% each). Lower extremity injuries comprised the remaining 36.4%, with ankle injury at 18.2% being the most common, followed by knee (9%) and foot (6%) injuries. Strains and sprains (42%) formed the bulk of the injuries, followed by fractures (21%), contusions (18%), and dislocation (9%). All pediatric athlete injuries were treated in the emergency department and released home, and none were admitted.
4. Squash:
  Squash, a well-known sport, is played on an enclosed court with a hollow rubber ball, and players must strike the wall; injuries can occur as the balls can reach speeds of 170 mph [1,12]. Horsley et al. [35] performed a retrospective analysis between 2004 and 2015 in 67 elite English players between 18 and 35 years. Most injuries were in the lower limb (76.5%), with the ankle and heel being the most common locations (20.8%), followed by the thigh (12.6%) and knee (10%). Most injuries were soft tissue injuries (71%). An overview of the type of injury revealed that tendonitis/bursitis was the most common (22.6%), followed by muscle spasm and muscle strain (16.2% each), ligament strain (9.6%), minor joint trauma (11.6%), atraumatic arthritis (7%), hematoma and chondral damage (5% each), and nerve damage and fracture (1% each). Another study by Rejeb et al. [36], looking at injury data from 166 adolescent multisport athletes from 2009 to 2014, showed that squash was the sport with the highest injury rate (8.5 injuries per athlete). The prevalence of overuse injuries was 50.3%, with most injuries involving the lower extremities (67%), of which the foot and ankle were the most common body parts. Genot Jendrusch [37] studied eye injuries due to high-velocity sports like squash, tennis, badminton in club, and school sports using the ARAG sport insurance database between 1987 and 2017 and found that 1.08% of injuries were eye injuries, out of which blunt trauma was more than 50% of cases.
5. Table tennis:
  Also called ping pong, it is a popular game played on a small table with a net, small racket bat, and soft hollow plastic ball. Studnicka and Ampat [38] described lumbosacral spondylolisthesis as common in children playing sports that require repetitive lower back extension, such as table tennis. They may present with lower back pain that worsens with activity. Tan et al. [39] analyzed a cohort of 2548 college students with primary anterior cruciate ligament (ACL) reconstruction in Hebei Province, China, and found table tennis, badminton, etc., to be the most prevalent sports causing ACL injuries. The patterns of ACL injuries were simplex ACL, meniscal injury, cartilage injury, and multi-ligament injuries.
6. Racquetball/paddleball:
  Racquetball is played similarly to squash but with a bigger court and no tin, and a larger and bouncier ball is used [1]. Changstrom et al. [40] performed a descriptive epidemiologic study of all racket sport injuries in US emergency departments between 2007 and 2016, and there were 75,615 injuries in people younger than 18 years. Tennis (69.9%) formed the bulk of their injuries, followed by squash/racquetball/paddleball (SRP) (16.5%) and badminton (6.4%). Lower extremity injuries accounted for 34% of all injuries, and upper extremity injuries accounted for 25%. Strains and sprains were the most common types of injuries at 39.8%, followed by fractures/dislocations at 18.6%. Cronin et al. [41] performed a multicentric orthopedic outcomes network (MOON) cohort study of 1235 patients (aged 12–66 years) with large labra tears of the shoulders and found racket sports among others like swimming, skiing, etc., (*p* = 0.01) to be associated with large labral tears.
7. Speedball:
  During this game, a racket must hit a ball tethered to a tall pole and it is famous in Egypt. Meshram et al. [42] conducted a descriptive epidemiological survey of 100 athletes between 18 and 41 years of age. In total, 65 out of 100 reported having at least one injury during the season, and most injuries occurred during practice (77%) rather than competition (23%). The most common sites of injury were the shoulder (50%), elbow (14%), and lower back (9%). The most common causes of injury were lack of training (40%) and lack of warming up (18%). Approximately 77% of them needed to visit a healthcare professional, and 9% needed surgery.

Table 2 summarizes most of these injuries and the sports that cause them.

## 4. Discussion

To our knowledge, very few studies have reviewed musculoskeletal injuries in the pediatric and adolescent populations in racket sports. This review shows that there are many studies involving tennis injuries, which show a wide variety of injuries with varying severity. Tennis has been shown to cause different types of injuries from concussion in the head, eye injuries, overuse of the shoulder, knee, back injuries, and fractures of the wrist, hand, knee, and rarely the rib [13,14,15,16,17,18,19,20,21,22,23,24,25,26]. This is understandable as tennis is a relatively fast-moving game that requires a powerful serve with an outstretched overhead hand movement. Johansson et al. [43,45] examined the association between spikes in external training load and shoulder injuries and back pain in competitive adolescent tennis players in his SMASH cohort study. The study found that accumulated external workload spikes of tennis training are associated with the higher rate of shoulder injuries and back pain and suggested that consistency in training load on a weekly basis is beneficial for reducing shoulder injuries rather than having a training schedule that comprises rapid increases in workload. Fernandez-Fernandez et al. [46] performed a randomized control trial to determine the influence of muscular fatigue and tennis serve performance within regular training sessions and examined 25 young male and females around 14 years of age who were given multiple training serve exercises before and after tennis training. It was found that it was more effective to provide serve training before the regular training of youth athletes; however, if it was given after training instead of before, there were excessive levels of fatigue, which could cause shoulder imbalances and increase the risk of injury. As per the rules of the International Tennis Federation, players must serve alternately from two different positions, the deuce on the right and the ad court on the left side. Fett et al. [47] studied the kinematic characteristic of the tennis serve and found that though the mean service velocity was similar on both sides, there were differences in the characteristics of the serve and ball kinematics. There were differences in the front dash foot angle relative to the baseline and lateral distance between the feet during the service. The upper torso range of motion from maximum clockwise rotation until impact was significantly greater on the deuce court. There is a benefit to understanding these differences and exploring adaptation in the service position by both players and coaches, which could help minimize injuries during the serve.

Badminton is a popular and easy game to play. Compared to tennis, badminton does not have a hard and heavy ball, but it has a shuttlecock made of feathers and is light. Although the speed of the shuttlecock can be fast, it has a low propensity to cause severe injury due to its lightness. We saw earlier in our results that most of the injuries related to badminton were in the lower extremities, which involved the knees and ankles, and most of the injuries were sprains and strains. However, due to the speed of the shuttlecock, rarely it can also cause injuries on the face, including ocular injuries. Zhao et al. [48] investigated the effects of integrated muscular training on injury prevention by enrolling 38 participants to high-risk and low-risk groups for 8 weeks of integrated neuromuscular training. It was found that integrated neuromuscular training can effectively improve the asymmetry of female athletes’ limbs, prevent sport injuries, and help improve their performance ability. Another study by Stausholm et al. [49] compared shoulder internal and external rotation strength in adolescent and adult elite badminton players. Adolescents demonstrated stronger shoulder external rotation bilaterally than adults, suggesting that increasing age may be associated with reduced shoulder muscle strength in elite badminton players. Although uncommon, rare case reports of humeral diaphyseal fractures due to badminton have also been reported [44].

Pickleball has emerged as a very popular sport in all age groups, which includes the pediatric population, over the last 5 years, as shown in the study by Boroumand et al. [34]. This study also showed that pediatric players had a higher frequency of upper extremity and finger injuries than adults. This could be attributed to jamming of the fingers during a fall [44] or the high end of the paddle handle for a better grip [50]. There were also more contusion injuries in the pediatric population, which could be attributed to poor muscle strength and coordination and incomplete mastery of the complex motor skills needed in a skillful game like pickleball [51]. This also highlights that warm-up and training before the game could help prevent injuries.

Squash is another popular racket sport, but it can lead to injuries as the ball travels very fast, and it is an enclosed game with multiple bounces that has a propensity to cause injuries in all parts of the body and sometimes cause serious injuries. Horsley et al. [35] showed that injuries were more prevalent in the lower age group of 18–23 years of age, which could be attributed to developmental changes occurring at a younger age, including changes in muscle strength, flexibility, and coordination [52]. Most injuries were also present in the lower extremities, such as the knee and ankle, which could be attributed to the rapid lunging and forward movements required in squash.

Table tennis or ping pong is another common racket sport enjoyed worldwide. There is a mix of fast play along with spinning, which requires delicate hand movements and first interactions with hand–eye coordination. There is repetitive overextension of the spine and back, which could lead to back injuries [38]. Due to its fast play, there is a possibility of lower extremity injuries, including knee injuries [39].

Racquetball and speedball are fast-moving games like squash and are known to cause injuries similar to those in squash. Most racquetball injuries are lower extremity injuries. Speedball, which originated in Egypt, is very popular in that area. Speedball-related injuries are commonly upper extremity injuries. Shoulder injuries can occur with too many serves or overheads in a short period of time, resulting in muscle fatigue and injury. Ankle and knee sprains occur due to a rapid change in direction, which could cause the angle to roll in and cause a sprain.

It may be interesting to compare these pediatric data with those of adult studies. Although there is no single review article comparing all racket sports data, we will review some important studies. Geoffrey Adams et al. studied tennis injuries and found that acute injuries like ankle sprains are common in the lower extremities while chronic overuse injuries like lateral epicondylitis are more common in the upper extremities [53]. Kondric et al. studied the frequency of injuries among Slovenian table tennis, tennis, and badminton players and found that the most injury-prone body parts were the shoulder girdle (17%), spine (16%), and ankle (15%). Most injuries occurred halfway through the training session or competition event, and injuries were mostly to muscle tissues, followed by joint and tendon injuries [54]. Neeru Jayanti et al. outlined that junior and elite players tolerate higher volumes and have more acute and lower extremity injuries and more serious overuse injuries, whereas adult recreational players tend to tolerate lower volumes, more overuse, and upper extremity injuries and have more conditions that are degenerative [12].

There is public health relevance in these review studies as they identify evidence gaps in the literature and combine all pediatric-related data across different racket sports. This is important because children and adolescents play racket sports in different places, such as schools and gyms, and injuries can cause not only pain but also loss of studies at school. Recovery from injuries can take time, increasing healthcare burden. More studies in the pediatric and adolescent population can lead to the creation of policies and prevention guidelines based on pediatric data instead of extrapolating from adult data.

One of the shortcomings of this review is that it is a narrative review; however, in the future, there could be room for more studies with well-defined systematic reviews of similar topics. One of the drawbacks of narrative reviews is the lack of statistical results, uneven studies, and selection bias compared to systematic reviews, which could be helpful in building better conclusions. There are more than 30 racket sports, but the lack of studies on less commonly played sports, including pickleball, in the pediatric population is surprising. There is a heavy dominance of sports in this review that have been popular for many decades, such as tennis and badminton. There could also be room for more kinematic studies of various sports in youth athletes involving children and adolescents, as this age group represents the age of growth and strength building. Another benefit of these kinematic studies could be in injury prevention, which is common at this young age group.

## 5. Conclusions

This is one of the few review studies on youth athletes’ racket sport injuries involving pediatric and adolescent populations that has covered all the available literature across racket sports, although data were absent for some racket sports. Tennis was the most studied sport among all racket sports, with a wide variety of injuries described. Overall, lower extremity overuse injuries were the most common injuries in most racket sports, followed by upper extremity injuries, which could be attributed to tennis being the most studied racket sport. Eye injuries were less common but serious. Most studies on children are limited to tennis, badminton, squash, and table tennis, which have a long history of being played, while there is a lack of studies on lesser-known racket sports, such as pickleball, which is only recently gaining popularity.

Youth athletes are more vulnerable to injury as they grow mentally and physically. More studies are needed to understand the sport kinematics of different racket sports, which could help prevent injuries. Injuries can cause not only pain but also time away from school/sports and increased healthcare utilization. Therefore, more studies on racket sport injuries could be beneficial in public health by decreasing school absences and hospital expenditures. The existing literature underscores the need for targeted injury surveillance and prevention strategies for pediatric and adolescent racket sport participants. There is also a need for more studies in the future, especially systematic reviews on racket sport injuries, to help bridge the gap in understanding them better.

## Figures and Tables

**Table 1:** Overview of the location of injuries and distribution of articles.

Location and Type of Injury	Number of Articles Describing These Injuries
Head	13 (total)
1.1. Eye injury	
1.1.1. Traumatic hyphema	2
1.1.2. Blunt eye injury	1
1.1.3. Macular hole	1
1.1.4. Other eye injury	7
1.2. Concussion	2
2. Back	6 (total)
2.1. Unspecified back injury	3
2.2. Lumbosacral spondylolisthesis	1
2.3. Lumbosacral injury	2
3. Upper extremity and chest	29(total)
3.1. Non-specific upper extremity injury	3
3.2. Shoulder injury	
3.2.1. Rotator cuff injury	1
3.2.2. Little league shoulder	1
3.2.3. Infraspinatus atrophy	1
3.2.4. Other shoulder injury	9
3.3. Rib injury-1st rib stress fracture	1
3.4. Arm injury	
3.4.1. Proximal humeral epiphysiolysis	1
3.4.2. Humerus diaphyseal fracture	1
3.4.3. Other arm injury	1
3.5. Clavicle fracture	1
3.6. Elbow injury	2
3.7. Trunk injury	1
3.8. Wrist/hand injury	
3.8.1. Non-specific injury	4
3.8.2. Metacarpal stress fracture	1
3.8.3. Carpal stress fracture	1
4. Lower extremity	39 (total)
4.1. Non-specific injury	8
4.2. Knee injury	
4.2.1. ACL injury	5
4.2.2. Patellar tendinopathy	1
4.2.3. Other knee injury	10
4.3. Ankle injury	11
4.4. Thigh injury	1
4.5. Hip injury	1
4.6. 5th metatarsal avulsion fracture	1
4.7. Sever’s disease (calcaneal apophysitis)	1
5. Miscellaneous	5 (total)
5.1. Sprain/strains	2
5.2. Overuse injuries	3

**Table 2:** Summary of injuries reported in racket sports.

Location	Injury	Racket Sports in Which Injury Is Reported	Notable Findings (If Present)
Head	Concussion [14,28]	Tennis (and other non-racket sports like track, lacrosse, soccer, etc.), badminton	Girls report more concussion per 10,000 exposures in major sports
Eyes	Traumatic Hyphema [13] Blunt injury to globe [13,32,33,37] Retinal detachment [32,33]	Tennis, badminton, squash	High number of hospitalized athletes due to tennis had open globe trauma
Chest/thorax	Stress fracture of the first rib [43]	Tennis	
Back and spine	Lumbosacral sprain [25,27,38] Lumbosacral spondylolisthesis [26]	Tennis, badminton, table tennis	
Shoulder	Overuse injuries, rotator cuff tendonitis, strain [16,17,30,34,42] Proximal humeral physis stress injury (Little League shoulder) [21] Infraspinatus atrophy [19] Fracture [18,34,40] Labral tears [41]	Tennis, badminton, pickleball, speedball	Overuse injuries common with medium- to high-velocity sports like tennis, badminton, squash, pickleball
Elbow	Tennis elbow, non-specific tennis injury [16,29,30]	Tennis, badminton	
Wrist and hand	Metacarpal stress fracture [31] Carpal stress fracture [18]	Badminton, tennis	
Arm	Humeral diaphyseal fracture [18,34,40,44]	Tennis, racquetball, pickleball, badminton	
Finger	Finger injury [17,34]	Tennis, pickleball	
Knee	Anterior cruciate ligament sprain or tear [22,27,28,34,39] Patellar tendinopathy [22]	Tennis, badminton, pickleball, table tennis	Knee is the most common site of overuse injuries
Foot and ankle	5th metatarsal avulsion fracture [22] Calcaneal apophysitis [24]	Tennis	

## Data Availability

Data are available upon request.

## Supplementary Materials

The following supporting information can be downloaded at: https://www.mdpi.com/article/10.3390/ijerph23010135/s1, Table S1: List of PMID of selected PubMed articles.

## Abbreviations

The following abbreviations are used in this manuscript:

SRC	Sport-related concussion
HSRIO	High school reporting information online
SMASH	Shoulder Management and Assessment Serving High Performance
MOON	Multicentric orthopedic outcomes network
