# Supplementary material for: Racket Sports-Related Injuries in Youth Athletes: A Narrative Review
Source: Int J Environ Res Public Health. 2026 Jan 22;23(1):135. doi: 10.3390/ijerph23010135 (PMC12840935; doi:10.3390/ijerph23010135)
Supplement: Supplementary file 1 [file ijerph-23-00135-s001.zip › ijerph-4037791-supplementary.pdf]

Table S1: List of PMID of selected PubMed articles

Number	Racket sport(s) involved and Year of publication	PubMed ID (PMID) of selected articles
1	Tennis (2025)	40320821
2	Overhead sports-Tennis (2025)	38898813
3	Pickleball (2025)	40254855
4	Squash, tennis, badminton (2025)	39809440
5	Badminton (2025)	39902842
6	Badminton (2025)	39843906
7	Table tennis (2023)	32809514
8	Tennis (2028)	30485004
9	All sports-Olympics (2024)	39628732
10	Tennis (2024)	39593526
11	Tennis, badminton (2024)	39049518
12	Badminton (2024)	39176324
13	Badminton (2024)	38643398
14	Major sports- Tennis, badminton etc. (2024)	36710411
15	Badminton (2024)	36533330
16	Tennis (2023)	37881190
17	Badminton (2023)	37761462
18	Badminton (2023)	36038652
19	Tennis (2023)	37325806
20	Badminton (2023)	36970317
21	Tennis (2023)	36057008
22	Tennis (2023)	36821418
23	Speedball (2022)	35468588
24	Tennis (2022)	35037501
25	Racket and paddle sport (2022)	33602024
26	Badminton (2022)	35647208
27	Badminton, tennis (2022)	34370212
28	Badminton (2021)	35198126
29	Badminton, tennis (2021)	33812701
30	Tennis (2021)	33460709
31	Badminton (2021)	33742024
32	Badminton (2021)	33469495
33	Squash (2020)	32823248
34	Tennis (2020)	31928289

35	Tennis (2020)	31662014
36	Tennis (2020)	31356120
37	Badminton (2020)	31796464
38	Badminton (2020)	30988018
39	Tennis (2019)	31311243
40	Tennis (2019)	30783695
41	Tennis (2019)	31561942
42	Tennis (2019)	31620485
43	Tennis (2019)	31366295
44	Tennis (2019)	29952177
45	Tennis (2019)	30702528
46	Tennis (2019)	29958840
47	Tennis (2017)	29316593
48	Tennis (2017)	29315449
49	Tennis (2017)	28351225
50	Badminton (2017)	27998001
51	Squash (2017)	28100124
52	Tennis, badminton (2017)	28387555
53	Tennis (2016)	26333792
54	Tennis (2016)	26647756
55	Tennis (2016)	26983458
56	Tennis (2016)	25944058
57	Tennis (2016)	26781295
58	Tennis (2015)	25872521
59	Tennis (2015)	26078449
60	Tennis (2015)	26491514
